# Resistance to Permethrin, β-cyfluthrin, and Diazinon in Florida Horn Fly Populations

**DOI:** 10.3390/insects9020063

**Published:** 2018-06-12

**Authors:** Chris J. Holderman, Daniel R. Swale, Jeffery R. Bloomquist, Phillip E. Kaufman

**Affiliations:** 1 Entomology and Nematology Department, University of Florida, Gainesville, FL 32611, USA; jbquist@epi.ufl.edu (J.R.B.); pkaufman@ufl.edu (P.E.K.); 2 Department of Entomology, Louisiana State University Agricultural Center, Baton Rouge, LA 70803, USA; dswale@agcenter.lsu.edu; 3 Emerging Pathogens Institute, University of Florida, Gainesville, FL 32611, USA

**Keywords:** acetylcholinesterase, insecticide, *Haematobia irritans*, *kdr*

## Abstract

Horn flies, *Haematobia irritans*, a major cattle pest in the USA, cause substantial economic losses and current control methods rely heavily on insecticides. Three horn fly populations were evaluated for insecticide susceptibility to permethrin, β-cyfluthrin, and diazinon. Susceptibility was variable by population, with the greatest resistance exhibited by a 66-fold resistance ratio (RR) to permethrin and >14-fold RR to diazinon. Mechanisms of resistance were determined using molecular techniques and enzymatic assays. The knockdown resistance (*kdr*) genotype (L150F) associated with pyrethroid resistance, and a G262A mutation in acetylcholinesterase, previously associated with organophosphate resistance, were found in all field populations evaluated. Insensitivity of diazoxon at the acetylcholinesterase (AChE) target site was significantly different in horn flies from one of the field sites. For metabolic detoxifying enzymes, cytochrome P450 nor general esterases showed a significant difference between field strains and a laboratory susceptible strain. Pyrethroid resistance was likely due to the presence of the L150F mutation in the population. In vitro studies targeting the AChE enzyme did not support the notion that the G262A mutation was the sole cause of resistance to organophosphates, and, therefore, the exact resistance mechanism to diazinon was not able to be confirmed.

## 1. Introduction

Horn flies, *Haematobia irritans*, are one of the most damaging pests of cattle in the United States. Each year they inflict over 876 million U.S. dollars in losses to U.S. cattle producers [1]. To curb these losses, horn flies are typically controlled by the use of synthetic insecticides applied to animal hosts [2], most commonly through the use of insecticide-impregnated ear tags. Resistance developed after the initial widespread use of ear tags in the early 1980s and has continued to the present [3].

Resistance in horn fly populations was first reported to the pyrethroid fenvalerate [4], and resistance was mitigated through the use of *S,S,S*-tributyl phosphorotrithioate (DEF) and piperonyl butoxide (PBO) [4], suggesting the resistance was due to an up-regulation of detoxification enzymes. Subsequent reports documented evolution of cross-resistance to other pyrethroids and DDT, and later, the identification of point mutations in voltage gated sodium channels (VgSC) [5,6,7]. Initially, metabolic synergists were added to formulations to overcome metabolic resistance mechanisms, but this method of resistance mitigation is ineffective in populations where target site mutations, such as knockdown resistance (*kdr*), are at high frequencies [8].

In horn flies, *kdr* and *super*-*kdr* are known to occur via the L150F and M54T mutations, respectively [6]. The *super*-*kdr* mutation is a second point mutation found in conjunction with *kdr* and is isolated to the VgSC. *Super*-*kdr* yields multiplicative resistance levels when compared to *kdr* and is therefore of critical importance to pest control programs. In the horn fly VgSC, *kdr* and the multiplicative *super-kdr* mutations confer reduced sensitivity to pyrethroids and are homologous to similar mutations described in *Musca domestica* L. (Wiedemann) [9]. Horn fly populations are often very difficult to reduce to below economic thresholds when *kdr* reaches high levels [2]. Currently, unknown mechanisms have been suspected of keeping *kdr* present in populations without selective pressures; in Chilean horn fly populations, where insecticide treatments had been discontinued for five years, *kdr* remained prevalent [10]. 

In addition to pyrethroid insecticides, horn flies have developed widespread resistance to organophosphate (OP) insecticides. However, after initial selection with diazinon ear-tags in Louisiana [11], there was cross-resistance to other, unused OPs (ethion, fenthion, pirimiphos-methyl), further complicating the development of successful control programs. Similar to pyrethroid insecticides, OP resistance is known to result from metabolic and target site mutations that reduce the bioavailability or potency at the target site, respectively. There are a number of mutations that have been shown to decrease OP sensitivity, but the G262A mutation in the horn fly acetylcholinesterase (AChE) enzyme is thought to be the primary mutation that confers reduced OP toxicity [12]. This mutation was evaluated by a multiplex PCR method that allowed for simultaneous identification of point mutations in both, the VgSC and AChE enzyme, which correspond to pyrethroid and OP target sites, respectively [13]. Several horn fly populations were evaluated and data suggested individual horn flies possessed both the G262A and the L150F mutations that likely yield OP and pyrethroid resistance, respectively [13].

The objectives of this study were to collect horn flies from the field and to quantify (1) insecticide susceptibility in treated jar assays against live flies, (2) confirm toxicity bioassay results with genetic prevalence of the G262A and the L150F point mutations in the AChE enzyme and VgSC, respectively, and (3) determine in vitro activity of general esterases and cytochrome P450s, which are known to metabolize pyrethroids and OP insecticides.

## 2. Materials and Methods

### 2.1. Insects

An insecticide susceptible horn fly colony (Kerrville), was obtained from New Mexico State University and originated from wild fly collections in 1961 at the USDA, ARS, in Kerrville, Texas [6]. Field collections of horn flies were accomplished by sweeping flies from the backs and bellies of mature cows and bulls at three sites: two University of Florida beef research sites, the Beef Teaching Unit (BTU) in Gainesville, FL; the Range Cattle Research and Education Center (Ona) near Ona, FL, and one privately owned herd located near Labelle, FL (Labelle). Each collection of flies was split between insecticide susceptibility assays and genetic/enzymatic assays. Horn flies for genetic/enzymatic assays were frozen, and subsequently dissected into the three major body regions (head, thorax, abdomen). The heads were utilized in AChE inhibition assays, the thoraces were utilized in genotyping, and the abdomens were retained for general esterase (CBE) activity or cytochrome P450 (cP450) assays.

### 2.2. Insecticide Susceptibility Assay

Field collected or laboratory colony horn flies were exposed to insecticide residues on glass, as has been previously described [14,15]. Briefly, technical grade insecticides; permethrin (47.6% *cis*:50.4% *trans*), β-cyfluthrin (99.5%), and diazinon (99.5%), were obtained from ChemService Inc. (West Chester, PA, USA). Serial dilutions of each insecticide in acetone (1 mL applied volume) were used to coat the insides of 60 mL glass jars [16], and jars were dried at room temperature for 24 h before use. Between nine and ten dilutions of each insecticide were used for each collection site. Immediately after collection in the field, adult male and female horn flies were knocked down with CO_2_ from a compressed gas cylinder and placed in groups of 15 in each jar. Because control mortality exceeded 20% at further time points, mortality at 4 hours was evaluated. Mortality was scored as an inability to walk, fly, or move. The assays were performed in the field, either under shade at ambient air temperatures or in the cab of an air-conditioned truck. The control treatment consisted of glass jars that had been treated as previously described with acetone only. Mixed sex, 2–5-day-old Kerrville flies were used as a susceptible reference strain to generate a concentration-response curve for all insecticides. Insecticide concentrations used to evaluate the Labelle flies were as follows; permethrin at 0.0902, 0.184, 0.368, 0.737, and 2.955 μg/cm^2^, β-cyfluthrin at 0.0147, 0.00737, 0.00147, 0.000147, and 0.0000147 μg/cm^2^, and diazinon at 0.0147, 0.0737, 0.147, 0.221, and 0.295 μg/cm^2^. Subsequently, a broader concentration range was used for horn flies from BTU and Ona as follows; permethrin, β-cyfluthrin, and diazinon were evaluated separately, each at 0.029, 0.295, 2.95, and 29.5 μg/cm^2^.

### 2.3. AChE Inhibition Assay

IC_50_ values (concentration needed to inhibit 50% of control enzyme activity) were determined using slight modifications of the Ellman protocol [17] outlined in Swale D.R. et al. [18]. Briefly, five frozen horn fly heads from each strain were pooled and homogenized in an electric motor driven tissue homogenizer in 0.1 M Na_2_HPO_4_ buffer (pH 7.8, Fisher Scientific, Pittsburg, PA, USA), 0.3% Triton X-100 (Fisher Scientific, Hampton, NH, USA), and Bovine Serum Albumin (BSA, 1 mg/mL, Fisher Scientific, Pittsburg, PA, USA). The homogenate enzyme solution (10 μL) was added to each well of a 96-well microassay plate, along with 20 μL of dissolved diazoxon and 150 μL of ice-cold phosphate buffer. The assay plate was incubated at 25 °C for 10 min. Ellman assay reagents Acetylthiocholine (0.4 mM final concentration) and 5,5′-dithiobis-(2-nitrobenzoic acid) (0.3 mM final concentration) were prepared fresh for each experiment, and 20 μL was added to the enzyme to initiate the reaction. Changes in absorbance were recorded by a DYNEX Triad spectrophotometer (DYNEX Technologies, Chantilly, VA, USA) at 405 nm. Seven inhibitor concentrations were used in triplicate to construct concentration–response curves using GraphPad Prism 4 (GraphPad Software, San Diego, CA, USA) that enabled interpolation of IC_50_ values. The final IC_50_ value resulted from the average of 3–5 IC_50_ determinations from each field site. Inhibitors were prepared using DMSO (dimethyl sulfoxide) and contained a final concentration of 0.1% DMSO (*v/v*) for each inhibitor concentration. Enzyme concentrations used were within the linear range of measured catalytic activity, thereby eliminating the need for protein quantification. IC_50_ values for each species were calculated by non-linear regression using PrismTM (GraphPad Software, La Jolla, CA, USA). 

### 2.4. Genotyping

At least 15 flies from each collection site were analyzed by PCR. Modifying the protocol outlined in Foil L.D. et al. [14], DNA was extracted from the thoracic regions of horn flies. Thoraces were pulverized using pre-chilled disposable pestles into 100 μL of sample buffer (100 mM Tris, pH 8.3, 500 mM KCl) within 1.5 mL pre-chilled microcentrifuge tubes kept on ice. Grinding of the fly thoraces continued until fully broken apart. Tubes containing ground thoraces were boiled at 100 °C for 3 min in a water bath. Tubes were centrifuged for 5 min at 15,000× *g*. One microliter of the supernatant was used in the PCR assay with a 1:1 (v:v) mixture of *AmpliTaq* DNA Polymerase (5 U/μL stock) and TaqStart Antibody (1.1 μg/μL stock), with the primer sets for resistant and wild type genotyping, and the PCR reagent concentrations as outlined in Jamroz R.C. et al. [19]. PCR amplification was completed in a Bio-Rad DNA Engine Peltier Thermal Cycler (Foster City, CA, USA) programmed for 96 °C for 2 min followed by 40 cycles of denaturation at 94 °C for 60 s, annealing at 62 °C for 1 min, and extension at 72 °C for 1 min, with a final extension at 72 °C for 7 min. Products were run on 3.0% agarose gel and visualized with ethidium bromide dye on a BioDoc-it Imaging System (Upland, CA, USA). If no amplification products were visualized after two attempts to amplify DNA, the samples were excluded from analysis, which occurred in 14% of samples. The PCR assay was conducted using a master mix of reagents was newly mixed for the samples run on a given day and was evaluated for contamination by loading a well of master mix in the thermal cycler with each run, and visualizing on a gel, as was done with the fly samples. Each reaction had the ability to generate three amplification products; a 154 bp GAPDH, glyceraldehyde-3-phosphate dehydrogenase, amplification product, a 285 bp L150F amplification product, and a 116 bp G262A amplification product. GAPDH is a constitutively expressed housekeeping gene that was used as a positive control in each reaction. The diagnostic L150F and G262A amplification product indicated either a susceptible or resistant gene was present in each fly [14]. 

### 2.5. cP450 and CBE Assays

Frozen horn fly abdomens were placed individually in a glass tissue homogenizer with 0.5 mL of 0.1 M sodium phosphate buffer (pH 8, Fisher Scientific, Pittsburg, PA, USA). The horn flies were homogenized with a motor-driven glass tissue homogenizer and centrifuged for 20 min at 12,000 rcf at 4 °C. The supernatant was utilized in both CBE and cP450 assays as the enzyme source.

The assay to determine cytochrome P450 (cP450) activity utilized the 1-step Slow TMB Substrate Solution Kit (Fisher Scientific, Fair Lawn, NJ, USA) following manufacturer’s instructions. Sample buffer (100 μL), 20 μL of enzyme source, and 80 μL of Slow TMB Substrate Solution Kit were added to each well of a 96-well microplate incubated for 1 h and absorbance read on the DYNEX *Triad* spectrophotometer at 620 nM. One enzyme homogenate was considered one replicate with at least three replicates performed from each horn fly collection site.

The abdomen homogenate from above was utilized as the enzyme source to measure general esterase activity [20]. In a 96-well microplate, 150 μL of sample buffer and 30 μL of substrate were added to each well. Substrate was generated fresh for each assay by mixing 1 mg of Fast Blue RR salt in 5 mL of sample buffer with 200 μL of 100 mM α-naphthyl acetate (α-NapA) dissolved in DMSO. A 20 μL aliquot of the abdominal enzyme source, described above, was added in triplicate to the plate. The plate was read immediately on the DYNEX *Triad* spectrophotometer at 595 nM. One enzyme homogenate was considered one replicate and at least three replicates were performed from each horn fly collection site. 

Protein levels were quantified for each enzyme source by utilizing the method described by Bradford M.M. [21]. Ten milligrams of Coomassie Brilliant Blue G (Sigma Aldrich, Saint Louis, MO, USA) dye was dissolved in 5 mL of ethanol, then suspended in a solution containing 8 mL of 10% HCl and 37 mL of deionized tap water. BSA (Sigma Aldrich, Saint Louis, MO, USA) stock was used as a standard by dissolving 1 mg into 0.15 M NaCl solution, and diluted serially into 0.15 M NaCl, generating 8 concentrations. The absorbance of BSA was read on the DYNEX *Triad* spectrophotometer (Chantilly, VA, USA) at 595 nm and used to construct a standard curve from absorbance values and known protein concentrations. The standard curve of BSA protein was compared to each fly sample, which allowed for the conversion of sample activity to a per fly abdomen unit of carboxylesterase activity. 

### 2.6. Statistics

Bioassay data from each horn fly strain were pooled and analyzed by probit analysis [22] using PoloPlus (LeOra Software, Petaluma, CA, USA) [23] for each insecticide to generate Lethal Concentration (LC) values and resistance ratio (RR) calculations. Abbott's transformation [24] for control mortality correction was applied within PoloPlus. Resistance ratios were generated by dividing the LC_50_ values obtained for horn flies from each collection site by the LC_50_ value obtained for the susceptible Kerrville strain.

Ellman assay absorbance net change was calculated for each strain, and analyzed by nonlinear regression to the following formula: Y = Bottom + (Top – Bottom) / (1 + 10 ^((LogEC_50_ – x)*Hillslope)); where the variable Bottom is the Y value at the bottom of the sigmoid (minimum value of 0), Top is the Y value at the top (maximum value of 100), x = the logarithm of the concentration and Y = the response (GraphPad Software, San Diego, CA, USA) [19]. Concentration-response curves were generated with GraphPad Prism and were utilized to determine IC_50_ values (concentration to inhibit 50% of enzyme activity), 95% confidence limits, Hill slopes, and R^2^ values. One enzyme source (5 fly heads per homogenate) and one dilution of anticholinesterase compound were considered one replicate. Each IC_50_ value was measured in triplicate using 7 concentrations. An average of at least nine IC_50_ values was generated from samples collected on separate days to obtain the reported IC_50_ value.

Average carboxylesterase activity and cP450 absorbance values per fly were compared by averaging replicates and an ANOVA was used to test differences between susceptible Kerrville flies and field collected flies in GraphPad Prism. The BSA standard curve generated a linear response (R^2^ = 0.99) that was used to back-calculate the amount of protein in each sample. The protein quantification was used to determine enzyme activity per unit and was converted to per milligram of abdominal protein.

## 3. Results

The susceptible Kerrville strain generated an LC_50_ value of 0.023 μg/cm^2^ for permethrin. Permethrin resistance ratios from field collections ranged from 3-fold with the Ona horn flies to over 60-fold greater with the Labelle horn flies (Table 1). In our evaluation of β-cyfluthrin, the susceptible strain had an LC_50_ value of 0.0067 μg/cm^2^. The field strains were variable in susceptibility to β-cyfluthrin, with the Ona population > 6.7-fold more susceptible and the BTU flies 1.4-fold less susceptible (resistant) when compared to the Kerrville strain. The highest evaluated dose of β-cyfluthrin (0.14 μg/cm^2^) resulted in 64% mortality for the Labelle horn flies but did not generate sufficient mortality across concentrations in order to provide an LC_50_ value. The Labelle horn fly strain was shown to be highly resistant to diazinon (>14.7-fold), but an exact value could not be calculated due to lack of homogeneity in field populations. Low numbers of collected flies prevented determination of resistance ratios to diazinon for the Ona population. Overall, sex of the evaluated wild-caught horn flies was not recorded and may have unintentionally impacted the results of the insecticide susceptibility assay.

**Figure 1:**
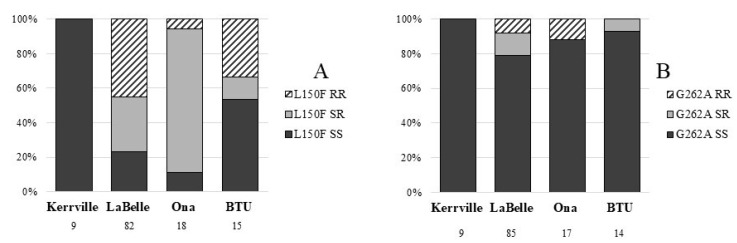
Percentage of horn fly resistant genotypes determined through a multiplex PCR. (**A**) illustrates genotyping for L150F, while (**B**) shows genotyping for G262A mutations. Laboratory strain = Kerrville (susceptible); field strains = LaBelle, Ona and BTU. RR = homozygous resistant, SR = heterozygotes, SS = homozygous susceptible. Number below fly strain name is the number of flies genotyped.

Genetic analysis using PCR showed the presence of the susceptible wild type *kdr* allele (L at position 150) in each horn fly tested from the susceptible Kerrville colony (Figure 1A). The Labelle horn flies contained 45% resistant L150F genotypes and 31% heterozygote genotypes. Ona horn flies showed 83% heterozygosity for the L150F genotype and the BTU horn flies were 53% wild type but had 33% homozygous L150F genotypes. The G262A mutation was found in each field strain evaluated but was absent in the laboratory strain. The greatest incidence of the G262A mutation was found in the Labelle horn flies with 13% heterozygous and 8% resistant horn flies identified. A smaller proportion of Ona and BTU flies was found with the G262A mutation. 

**Figure 2:**
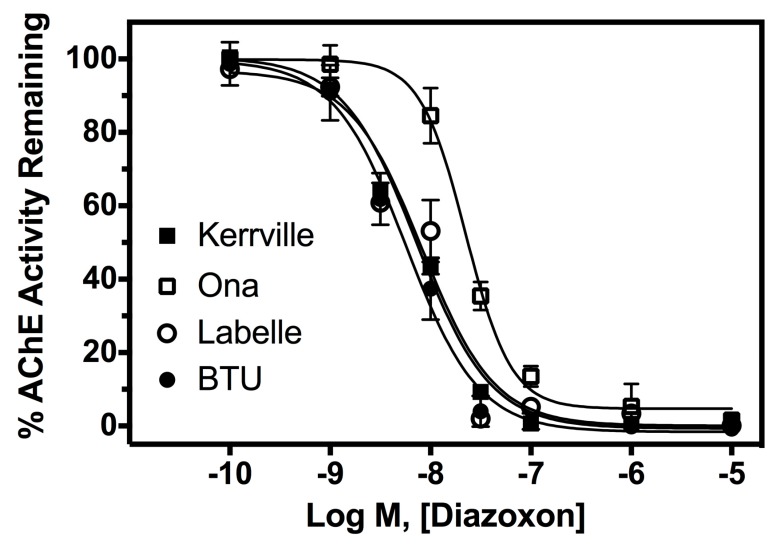
Horn fly head homogenate AChE inhibition (IC_50_) by diazoxon, showing significant differences between the Ona horn flies and others. Laboratory strain = Kerrville (susceptible); field strains = LaBelle, BTU and Ona.

In vitro potency assays were used to determine the affinity of diazinon to wildtype and field collected horn fly AchE (Figure 2). The IC_50_ value of diazoxon for the Kerrville (susceptible) population was found to be 8.2 nM (95% CI: 6–10 nM; Hillslope: −1.0; r^2^: 0.99). The IC_50_ values of diazoxon for the Labelle and BTU populations were found to be not statistically significant when compared to Kerrville with IC_50_ values of 9.2 nM (95% CI: 5–15 nM; Hillslope: −1.1; r^2^: 0.99) and 6.1 nM (95% CI: 5–7 nM; Hillslope: −1.1; r^2^: 0.99), respectively. However, the Ona population was found to be significantly less sensitive (*p* < 0.01) to diazoxon inhibition when compared to Kerrville, Labelle, and BTU populations, having an IC_50_ value of 20.5 nM (95% CI: 18–23 nM; Hillslope: –2.2; r^2^: 0.99). 

Unfortunately, due to difficulty in collecting adequate numbers of horn flies, the Ona population was not evaluated in the jar assay. Thus, we have no definitive evidence for resistance to diazinon through AChE insensitivity, as we only have molecular genotyping that is not coupled with live insect bioassay data. Additionally, no statistical differences were found in the cP450 assay conducted in these strains (F = 0.93, df = 3,16, *p* = 0.44). Overall, general esterase assay did not differ across strains (F = 2.69, df = 3,16, *p* = 0.08) (Table 2).

## 4. Discussion

The Kerrville strain was previously evaluated for sensitivity to diazinon residues on glass, generating an LC_50_ value of 2.0 μg/cm^2^ in a 2-h exposure [25], which is 100-fold greater than our results. This difference was likely due to the shorter assay duration, as diazinon must be bioactivated to diazoxon in the insect prior to becoming neurotoxic [26]. We did not evaluate for recovery, which may have occurred if our assay was extended longer than four hours. Laboratory experiments in house fly have previously shown insecticide resistant mutations have fitness costs, and colonies maintained for several generations will change the proportion of resistant alleles, based upon the fitness costs of each mutation [27]. However, this finding stands in contrast to previous field studies on horn flies, where after several years of no insecticide applications, *kdr* remained present in the population [10]. 

Due to the difficulty in collecting adequate numbers, not all field strains could be evaluated with all insecticides. The RRs for all insecticides at the LC_50_ values were lowest for the University of Florida properties, BTU and Ona.

Product (insecticide-impregnated ear tag) failure was reported by the private herd owner at the LaBelle site, and is supported in our evaluation using the glass jar assay and high resistance ratios found for permethrin and diazinon. The owner did not have detailed records on insecticide application practices. The RR was 66-fold for permethrin but could not be determined for the evaluated concentrations of β-cyfluthrin. Comparatively, the RR for permethrin has been reported as high as 54 in Georgia, USA horn fly populations [28]. β-cyfluthrin has no previously published LC_50_ values with horn flies. Additionally, at the Labelle field site, we were unable to generate LC_50_ values for diazinon due to lack of mortality at the concentrations tested; our estimated RR is greater than 14.7. Previous resistance to diazinon has been reported with RR of up to 55-fold in an 18 h exposure on cloth [29], 7.7 in a 4-h exposure on filter paper [11], and 1.2 in a 2-h exposure on filter paper [30].

Mechanisms of horn fly resistance have been previously reported as target site-mediated via L150F and G262A mutations, and detoxification related via cP450 and general esterases [8]. The present study documented that the LaBelle collection had resistant L150F alleles in 75% of the population, while the suspected AChE insensitivity mutation was only found in 20% of the population. The high values for the resistance-coding L150F alleles indicates this mode of resistance likely is responsible for conferring resistance to pyrethroids. Organophosphate resistance was not explained by the presence of the G262A mutation or diazoxon inhibition potency in ACHE assays. Both Ona and LaBelle populations were determined to have G262A mutations, but only the Ona flies showed differences in IC_50_ values. However, because the AChE inhibition assays required a pooled sample, the assay may not have been sensitive enough to quantify differences between samples. Susceptible field populations of horn flies were observed to have similar rates of G262A mutation as the resistant collections (Figure 1B), suggesting additional factors may be involved. Previously, the G262A mutation has been reported at low levels (7.5–23.8% of resistant genotype) for at least 5 years without direct application of organophosphates to cattle [31]. The authors evaluated resistance mechanisms in a multiplex PCR for presence of G262A, *kdr* and *rdl*. The study did not evaluate tolerance or resistance to insecticides in an exposure assay but does suggest little or no fitness costs for the G262A mutation. Our finding of G262A in populations without resistance in exposure assays was unexpected and warrants further investigation. Previous studies suggest that secondary mutations are responsible for conferring resistance to organophosphates in the presence of the G262A mutation, as is the case in other insects [31].

Other mechanisms that may have conferred resistance include GSTs and penetration resistance [32], both of which were not evaluated. GSTs have been documented as a cause of resistance, specifically to OPs, in several diverse insect orders, including Hemiptera [33], Orthoptera [34], and Lepidoptera [35]. Evaluation of GSTs could not be completed due to the difficulty in collecting horn flies.

During the course of this research, several problems were encountered with adequate fly collection numbers. For example, Ona flies were not collected in sufficient numbers to evaluate contact resistance to diazinon, yet potency of diazoxon to AChE of Ona flies was significantly reduced. Future researchers should strongly consider the utilization of a discriminating dose for field populations in contact assays, rather than attempting to determine LC_50_ values. Had such procedures been undertaken, the collected numbers of flies would have been sufficient for analysis. Population level fitness costs have been evaluated previously in horn flies [36], and consideration should be given to future projects that evaluate population level evolution to resistance. Furthermore, we evaluated for only one known mutation and others may have existed in the evaluated populations, if fewer flies were used for jar assays in future studies, additional samples could be retained for mutation screening.

Horn fly control is important to reduce cattle production inputs and maintain profitability for cattle producers. The present study documented a Florida population of horn flies with very high levels of resistance to two insecticide classes from the very limited group available for cattle producers. Resistance to insecticides has been documented numerous times in the literature for horn flies [8] and our research continues to show the need for new chemistries and insecticide-alternatives for this production system. Negative cross-resistance has been suggested as a management practice for organophosphate and pyrethroid insecticides [25,35,37]; however, our results document a horn fly population capable of resistance to both diazinon and permethrin (LaBelle). Lack of negative cross-resistance has previously been reported for horn fly populations with RR to diazinon, permethrin, and λ-cyhalothrin in Louisiana [11]. Future efforts to control horn flies should focus on insecticide resistance mitigation practices and development of novel classes of insecticides.

## Figures and Tables

**Table 1:** Toxicity of permethrin, β-cyfluthrin and diazinon on glass to horn fly adults collected from Florida beef cattle ranches.

Site	Permethrin				β-cyfluthrin				Diazinon			
	LC_50_ value ^A^	95% CI	Slope	RR ^B^	LC_50_ value	95% CI	Slope	RR	LC_50_ value	95% CI	Slope	RR
Kerrville	0.023	0.016–0.032	2.47		0.0067	0.0044–0.0096	1.55		0.02	0.14–0.029	2.45	
Labelle	1.53 *	1.01–2.6	1.29	66.6	ND ^C^	ND	ND	ND	>0.295 ^D^	ND	0.158	>14.7
BTU	0.13 *	0.055–0.19	1.36	5.7	0.01	0.0013–0.13	0.79	1.4	0.06 *	0.046–0.27	1.33	3
Ona	0.068	0.013–0.36	0.85	3.0	<0.001	ND	ND	<0.15	ND ^E^	ND	ND	ND

^A^ LC_50_ is in μg/cm^2^, ^B^ resistance ratios were calculated as LC_50_ of the field strain/LC_50_ Kerrville strain, ^C^ the highest tested concentration of 0.14 μg/cm^2^ generated 64% mortality, ^D^ the highest tested concentration of 15 μg/cm^2^ generated 20% mortality, ^E^ Not determined due to difficulty in collecting enough flies to evaluate in the assay. ND = not determined. Asterisk (*) indicates statistical significance when 95% CI do not overlap with the susceptible strain. The total number of flies tested for Kerrville was n = 900, other fly strains n~300 for each insecticide listed. Due to difficulty in collecting wild flies not all sites could be evaluated for each insecticide. Numbers after < or > indicate that the tested values did not generate a LC_50_ value because the doses did not bracket the LC_50_, did not conform to heterogeneity, or other statistical requirements for probit analysis; the difficulty in collecting and limited numbers of field-collected horn flies prevented further replicates. Approximately 40–50 field collected horn flies were evaluated in each concentration of insecticide.

**Table 2:** Enzymatic activity ± SEM per milligram of horn fly abdomen protein.

Collection Site	Cytochrome P450	General Esterase
Kerrville	29.1 ± 8.9	13.5 ± 5.4
Labelle	50.5 ± 8.22	40.6 ± 10.3
Ona	23.3 ± 10.1	23.0 ± 4.5
BTU	32.1 ± 18.4	32.3 ± 6.8
ANOVA		
F-Value	0.93	2.69
*p*-Value	0.44	0.08

Enzymatic activity was defined as absorbance divided by calculated protein in each sample. Average carboxylesterase activity and cP450 absorbance values per fly were compared by averaging replicates and an ANOVA was used to test differences between means of each strain. The BSA standard curve generated a linear response (R^2^ = 0.99) that was used to back-calculate the amount of protein in each sample. The protein quantification was used to determine enzyme activity per unit and was converted to per milligram of abdomen activity. Enzyme activity was scaled to a per milligram basis. Five replicates were averaged for each collection site. ANOVA was performed on the results and no significant differences were found, df = 3,16.
